# Relationship between Telework Jetlag and Perceived Psychological Distress among Japanese Hybrid Workers

**DOI:** 10.3390/clockssleep5040040

**Published:** 2023-10-16

**Authors:** Yuuki Matsumoto, Ayako Hino, Kunitaka Kumadaki, Osamu Itani, Yuichiro Otsuka, Yoshitaka Kaneita

**Affiliations:** 1 Department of Nursing, School of Medicine, Kurume University School of Nursing, Kurume 830-0003, Japan; 2 Division of Public Health, Department of Social Medicine, Nihon University School of Medicine, Tokyo 173-8610, Japan; 3 Department of Mental Health, Institute of Industrial Ecological Sciences, University of Occupational and Environmental Health, Kitakyushu 807-8555, Japan; 4 Department of Internal Medicine, Univer sity of Occupational and Environmental Health, Kitakyushu 807-8555, Japan

**Keywords:** telework, social jetlag, sleep, midsleep point, psychological distress, mental health, Japanese

## Abstract

Social jetlag is associated with physical and mental health problems. With the increased popularity of telework, we investigated a specific form of social jetlag that we termed “telework jetlag”. This study aimed to clarify the relationship between telework jetlag—the difference in sleep and wake-up times between in-office and telework days—and mental health problems among Japanese hybrid workers. A cross-sectional study was conducted with 1789 participants from October to December 2021 using an online-based questionnaire. Telework jetlag, defined as the difference in the midsleep point between in-office and telework days, was investigated using two groups according to telework jetlag—those lagging <1 h versus ≥1 h. We used the six-item Kessler Scale as a nonspecific psychological distress scale for the outcome. Telework jetlag was significantly associated with psychological distress, and the ≥1 h group had a higher risk (odds ratio: 1.80) of developing high psychological distress (HPD) than the <1 h group in the multivariate analysis. Since most teleworkers are forced to have a hybrid work style that mixes going to work and teleworking, telework jetlag must be addressed to maintain the health of teleworkers.

## 1. Introduction

Social jetlag refers to the difference in sleep timing (the times of going to bed and waking up) between duty days (days when individuals must go to school or work) and free days when the two alternate [1]. On free days, people usually wake up at the time of their choice, while on duty days, they must wake up according to work or school start times. In particular, those who live far from their workplace or school wake up earlier to accommodate travel time. Besides that, social jetlag is also associated with various lifestyle factors such as smoking, caffeine intake, alcohol consumption, and exercise [1,2], as industrialized countries, more than 70% of the population has over 1 h of social jetlag [1,3,4]. Social jetlag is associated with physical and mental health problems such as obesity, metabolic diseases, malignant tumors, and psychiatric disorders resulting from the disruption of circadian rhythms [4,5,6,7,8,9,10,11]. These mechanisms involve abnormal rhythms of glucocorticoid secretion and postprandial glucose response during the internal desynchronization of circadian rhythms [12], abnormal diurnal rhythms of gene expression [13], stress responses, such as elevated blood cortisol levels and resting heart rate [2], and mood instability [14].

A new way of working has become popular since the outbreak of the COVID-19 pandemic in 2020: teleworking. Telework eliminates commuting time and thus decreases social jetlag [15,16,17,18,19]. Telework is expected to contribute to not only convenience, but also the prevention of social jetlag.

However, there is concern that those who partake in teleworking, a phenomenon which spread rapidly after the COVID-19 pandemic, have inadequate knowledge of how to maintain good health [20,21]. Some studies have reported that teleworking could have various effects on mental health, such as increased stress due to the difficulty of separating work and personal life and social isolation due to the lack of face-to-face interactions [22,23,24,25,26,27,28]. Individual differences in the benefits and harms of telework have also been reported, and various work conditions such as telework frequency, location, social support, etc., may cause them [29].

In addition to those issues, we are concerned about the potential for new work styles, including telework, to cause a new jetlag. Few jobs can be completed entirely via telework, and almost all people must visit the workplace several times each week or month. Therefore, most teleworkers have a hybrid work style that combines going to the workplace and teleworking. We named the resulting difference in sleep timing between in-office and telework days “telework jetlag”. If social jetlag can cause health problems, perhaps telework jetlag can as well.

To our knowledge, no studies have investigated the relationship between telework jetlag and mental health problems among teleworkers. In the period when early telework studies were reported, the Wi-Fi environment was not as prevalent as it is today, and the telework environment could be quite different [25,30,31]. In Japan, especially, teleworking was not widespread before the COVID-19 pandemic, and there are a few studies on sleep problems and mental health among Japanese teleworkers. We hypothesize that mental health problems for workers with a hybrid work style, mixing going to work with telework (hybrid workers), are associated with telework jetlag. This study aimed to clarify the relationship between telework jetlag and mental health problems among Japanese hybrid workers.

## 2. Results

### 2.1. Participant Characteristics

Responses were obtained from 2032 participants. Of these, 11 individuals who did not consent to participate in the study, 8 who had been on leave within the past month, and 224 who had not teleworked within the past month were excluded, leaving 1789 (1231 men and 558 women; mean age, 43.2 years; standard deviation, 11.3) individuals who were included in the analysis. The valid response rate was 60.2%. Table 1, Table 2 and Table 3 present the participants’ characteristics. There were 232 participants (13.0%) with a telework jetlag of 1 h or longer. Regarding psychological distress, 1524 (85.2%) patients were classified as having non-high psychological distress (NHPD), and 265 (14.8%) were classified as having high psychological distress (HPD).

### 2.2. Prevalence of HPD for Telework Jetlag

Table 4 shows the aggregate results based on psychological distress and telework jetlag. A higher percentage of HPD (22.0) was found in participants with ≥1 h of telework jetlag, and there was a significant difference in the χ2 test.

### 2.3. Characteristics of Participants Divided by Telework Jetlag

**Figure 1:**
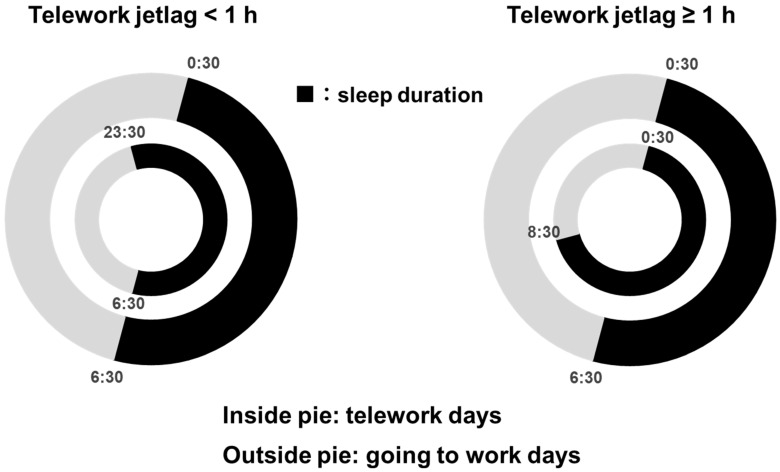
Median times of going to bed and waking up for telework and in-office days divided by telework jetlag.

Figure 1 shows the median times of going to bed and waking up on telework and in-office days for two groups based on telework jetlag. Both groups slept longer on the telework days; however, the <1 h group went to bed 1 h earlier, while the ≥1 h group woke up 2 h later.

### 2.4. Odds Ratio of HPD in Telework Jetlag

The multivariate regression results are presented in Table 5. The input covariates in each model were as follows: Model 1: sex, age, living alone, telework frequency, telework location, and telework duration; Model 2: occupation, employment position, work hours in the past month, job demands, job control, supervisor support, coworker support, and all covariates of Model 1; Model 3: commuting time, means of commuting, exercise habits, drinking alcohol, consuming caffeine, current smoking status, using electronic terminal outside work, enjoying leisure time, and all covariates of Model 2; and Model 4: sleep duration, Athens Insomnia Scale score, and all covariates of Model 3. In all of the analysis models, the results of the suitability tests were good (i.e., omnibus test: *p* < 0.05; Hosmer–Lemeshow test: *p* ≥ 0.05). Telework jetlag was significantly associated with psychological distress, and the ≥1 h group had a higher risk of developing HPD than the <1 h group in all analysis models. The results for other covariates (*p*-value and 95% CI) are presented in the Supplementary Material (Table S1).

## 3. Discussion

In order to establish telework as a sustainable work style, many research studies and policies that contribute to the reduction in health risks due to telework and the promotion of workers’ health are required [32,33]. This study examined the relationship between various telework jetlag and psychological distress among hybrid workers. Telework jetlag was significantly associated with psychological distress even after adjusting for confounding factors such as telework status, lifestyle habits, job stress, and sleep duration. The strength of this study is that it defined the concept of telework jetlag and found that different times of going to bed and waking up on in-office and telework days are associated with psychological distress. Telework jetlag may be a risk factor for mental health problems among hybrid workers, which is important to consider when promoting health management and health promotion among hybrid workers.

First, we discuss the characteristics of the sample population based on the outcome variables. While 10.3% of those aged 20 or above in the 2019 Comprehensive Survey of Living Conditions had six-item Kessler Scale (K6) scores ≥ 10, the percentage was 14.8% for the participants in this study [34]. Thus, the results of this study indicate a higher rate than the national average. However, as 2019 was before the COVID-19 pandemic, it is difficult to compare the results.

Next, for the results of multivariate regression, in Model 1, telework jetlag was found to be significantly associated with psychological distress, even after adjusting for telework frequency, location, and duration. This means that hybrid workers should be aware of the presence of telework jetlag regardless of these various telework statuses. Since telework jetlag is an original conception defined in this study, the factors associated with it are not clear; nevertheless, it is possible that the likelihood and influence of telework jetlag may differ depending on the occupation and work environment (job demand, job control, and social support). The results of Model 2, however, showed that there was still an association between telework jetlag and psychological distress even after adjusting for the occupation and work environment. These findings mean that telework jetlag should be noted regardless of occupation or work environment for the management of mental health in hybrid workers. Furthermore, in Model 3, telework jetlag was significantly associated with psychological distress, even after adjustment for various lifestyle factors. As mentioned earlier, social jetlag is associated with various lifestyle habits, which are also risk factors for mental health problems [35,36,37]. Therefore, the results of Model 3 suggest that telework jetlag is associated with psychological distress independently of such lifestyle habits. On the other hands, since it was pointed out that social jetlag is affected by a lack of sleep, it is recommended to correct for sleep duration [38,39,40]. However, the results of the multivariate analysis showed that the 95% CI range of Model 4 (adjusted for sleep duration) and Model 3 (unadjusted for sleep duration) remained stable. Therefore, the effect of sleep duration on telework jetlag was considered to be small. Okajima et al. stated that although social jetlag and sleep deprivation are positively correlated, their association is small and they are separate concepts [41]. The results of this study on telework jetlag also support their findings.

We also discuss the possible biological mechanisms by which telework jetlag is associated with psychological distress. It has been reported that in social jetlag, the delayed onset of melatonin causes fatigue and drowsiness that persist for several days [42]. Therefore, the disturbance of the melatonin secretion rhythm may also occur in telework jetlag, which may be related to mental condition. A meta-analysis of prospective studies showed an increase in depression among shift workers with disrupted circadian rhythms [43]. A disruption of the circadian rhythm is an important risk factor for mental disorders such as depression [44,45]. The biological mechanism is that irregular sleep timing alters the secretion patterns of hormones, such as melatonin, cortisol, and noradrenaline, which contribute to the development of depressive symptoms [12,46,47,48,49]. In particular, the wake-up times differed by 2 h between the in-office and telework days in the ≥1 h group. Since morning sunlight upon waking is a major synaptic signal for circadian rhythms, changes in wake-up times could greatly affect circadian rhythms [50,51]. However, the participants in the <1 h group woke up at the same time on in-office and telework days and went to bed 1 h earlier. Therefore, they were able to obtain sleep duration with less impact on their circadian rhythms than the ≥1 h group.

However, there is a major limitation to our discussion. If the K6 score is ≥10, a pathological mental disorder that requires medical attention is suspected. Abnormal deep body temperature rhythms and the over-secretion of cortisol, melatonin, and so on often appear in patients with depression [52,53,54,55,56,57]. Therefore, it is possible that telework jetlag is caused by symptoms of mental illness. Since this was a cross-sectional study, it was not possible to prove the temporality of the association. A longitudinal analysis is needed in the future.

Other limitations of this study are as follows: First, there is a possible selection bias because the study only included workers of companies in Tokyo, the capital of Japan. Given Tokyo’s high population density and advanced communications technology, it may have a different telework environment than rural areas. Second, because the survey was entirely self-reported and subjective, possible information bias may have made the responses less objective. Finally, there could be confounding bias due to factors that were not entered as covariates, such as household income and medication history. In particular, those with restricted employment may disproportionately belong to the HPD type.

## 4. Conclusions

We defined telework jetlag, which is a new concept that differs from social jetlag, as the difference in sleep timing between in-office and telework days. This study showed that telework jetlag of 1 h or more was significantly associated with mental health problems. Telework jetlag may be a risk factor for mental health problems among hybrid workers, which is important to consider when promoting health management and health promotion among hybrid workers. Since most teleworkers are forced to have a hybrid work style that mixes going to work and teleworking, telework jetlag must be addressed to maintain their health. In particular, the delay in wake-up time may need to be focused on.

Our study will contribute substantially to improving the health of teleworkers and making this work style sustainable. However, future longitudinal studies are needed to establish a causal relationship between telework jetlag and mental health problems.

## 5. Materials and Methods

### 5.1. Study Design, Participants, and Ethical Considerations

A cross-sectional study using an online-based questionnaire was conducted from October to December 2021. The participants were 2971 day shift workers employed by four companies in Tokyo who consented to participate in the survey. Since the population of Tokyo is approximately 10 million, we considered a sample size of at least 1067 persons to be sufficient, assuming a 95% confidence level, a 3% margin of error, and a population ratio of 0.5. The purpose and procedures of the study were explained to the employees. Participation was voluntary, and we explained that no disadvantages would arise from not participating in the study and that the data obtained would not be used for any purpose other than the study.

Consent for participation was obtained through a web-based response before participation, and those who did not agree were not able to access or respond to any of the research items. Since this was an anonymous study with a substitute employee ID number, they were also told that it was possible to withdraw consent after answering the study and that the responses would be deleted if consent was withdrawn. This study was conducted in accordance with the Declaration of Helsinki and its current amendments. It was approved by the Ethics Committee of the Nihon University School of Medicine (approval no. 2021-02).

### 5.2. Telework Jetlag and Other Telework Status

To calculate telework jetlag, we referred to the social jetlag calculation method [1]. We first determined the midsleep points on in-office and telework days. The midsleep point is the median value between the times of going to bed and waking up (e.g., if a person goes to bed at midnight and wakes up at 6:00 am, the midsleep point is 3:00 am). The items regarding the times of going to bed and waking up were phrased as follows: “What time did you (go to bed/wake up) on (in-office/telework days) in the past month?” The difference in midsleep points between in-office and telework days was defined as telework jetlag. Since social jetlag of 1 h or more was reported to be associated with health problems [6], participants in this study were also classified according to telework jetlag as <1 h or ≥1 h.

The item regarding teleworking frequency was “How much did you telework in the past month?” The choices were categorized using a five-answer method: (1) not teleworking, (2) 1 to 3 times per month, (3) 1 to 2 times per week, (4) 3 to 4 times per week, and (5) ≥5 times per week. The item regarding telework location was “Please select one location where you telework most frequently”. The choices were categorized using a four-answer method: (1) living room, (2) private room, (3) bedroom, and (4) other. The item regarding teleworking duration was “How long have you been teleworking?” The choices were categorized using a three-answer method: (1) <1 y; (2) ≥1 y and <1.5 y, and (3) ≥1.5 y. These telework statuses were entered as covariates when performing the multivariate analysis.

### 5.3. Psychological Distress (Outcome)

We used the K6 as a nonspecific psychological distress scale for outcomes [58]. The K6 was developed to screen for depression, anxiety disorders, and other mental health conditions, and the reliability and validity of the Japanese version were established [59,60]. It can be used in surveys of the general population and is widely used as an indicator of the degree of psychological stress. The cutoff values of the K6 are 4/5 and 9/10 points. In this study, a score of ≤9 was defined as indicating NHPD, and a score of ≥10 was defined as indicating HPD.

### 5.4. Other Covariates

Basic attributes, lifestyle habits, occupational stress, and insomnia were entered as covariates. The basic attributes included sex, age, presence of a roommate, occupation, employment position, commuting time, means of commuting, and work hours in the past month. Lifestyle habits included exercise, alcohol consumption, caffeine intake, current smoking habits, use of electronic terminals outside of work, enjoyment of leisure time, and sleep duration. Occupational-stress-related factors were adapted from the Brief Job Stress Questionnaire and included job demands, job control, supervisor support, and coworker support [61]. Insomnia was measured using the Athens Insomnia Scale (AIS). The AIS was developed in accordance with the diagnostic criteria for insomnia in the International Classification of Disorders, and its Japanese version was verified and validated by Okajima et al. [62,63]. The questionnaire consists of eight items with a score range of 0–24 points. The cutoff value is 5/6 points, with higher scores indicating stronger insomnia symptoms.

### 5.5. Statistical Analysis

The χ2 test was used for univariate analysis in cross tabulations. Multiple logistic regression was used for multivariate analysis, and odds ratios and 95% confidence intervals were calculated using the direct method. Omnibus and Hosmer–Lemeshow tests were used to assess the suitability of the analysis model. Statistical analyses were performed using SPSS for Windows Version 28.0. All tests were two-tailed with *p*-values < 0.05 noting statistical significance.

## Figures and Tables

**Table 1:** Characteristics of participants regarding basic attributes.

Characteristics			*n*	%
	Sex			
		Men	1231	68.8
		Women	558	31.2
	Age			
		20–29	285	15.9
		30–39	389	21.7
		40–49	477	26.7
		50–59	537	30.0
		≥60	101	5.6
	Living alone			
		No	1302	72.8
		Yes	487	27.2
	Occupation			
		Clerks	337	18.8
		Engineers	379	21.2
		Field workers	67	3.7
		Creators	115	6.4
		Customer service	69	3.9
		Sales	716	40.0
		Other	106	5.9
	Employment position			
		Manager or director	114	6.4
		Chief	341	19.1
		Supervisor	266	14.9
		Regular employee	1068	59.7
	Commuting time			
		<1 h	1130	63.2
		≥1 h	659	36.8
	Means of commuting			
		Crowded train	961	53.7
		Uncrowded train	453	25.3
		On foot	189	10.6
		Other	186	10.4
	Work hours in the past 1 month			
		<160 h/m	515	28.8
		≥160 and <200 h/m	954	53.3
		≥200 h/m	320	17.9
Lifestyle			*n*	%
	Exercise habits			
		Not physically active and no intention to change	172	9.6
		Not active but intending to change	442	24.7
		Perform some activity but not enough	643	35.9
		Regularly active but not in the habit	235	13.1
		Regularly active in the habit	297	16.6
	Drinking alcohol			
		Never or rarely	611	34.2
		Less than 2 d/wk	484	27.1
		3–6 d/wk	364	20.3
		Every day	330	18.4
	Consumption of caffeine			
		Never or rarely	155	8.7
		2–6 d/wk	331	18.5
		Every day but less than twice a day	826	46.2
		Every day and more than three times a day	477	26.7
	Current smoker			
		No	1321	73.8
		Yes	468	26.2
	Using electronic terminal outside of work			
		<2 h/d	860	48.1
		≥2 h/d	929	51.9
	Enjoying leisure time			
		Yes	1171	65.5
		No	618	34.5
	Sleep duration			
		≥7 h	1108	61.9
		<7 h	681	38.1

**Table 2:** Characteristics of participants regarding telework jetlag and various telework statuses.

Telework Frequency	*n*	%
1–3 d/m	321	17.9
1–2 d/wk	436	24.4
3–4 d/wk	626	35.0
≥5 d/wk	406	22.7
Telework location		
Living room	843	47.1
Private room	410	22.9
Bedroom	409	22.9
Other	127	7.1
Telework duration		
<1 y	253	14.1
≥1 y and <1.5 y	566	31.6
≥1.5 y	970	54.2
Telework jetlag		
<1 h	1557	87.0
≥1 h	232	13.0

**Table 3:** Characteristics of participants regarding each scale.

	*n*	%	Mean	SD
Psychological distress				
NHPD (K6 score < 10)	1524	85.2		
HPD (K6 score ≥ 10)	265	14.8		
AIS score				
<6	1150	64.3		
≥6	639	35.7		
Job stressors and social supports				
Job demands			8.6	1.9
Job control			8.7	1.7
Supervisor support			8.1	2.2
Coworker support			8.4	2.1

**Abbreviations:** SD: standard deviation, NHPD: non-high psychological distress, HPD: high psychological distress, AIS: Athens Insomnia Scale.

**Table 4:** Cross-tabulation and χ^2^ test based on psychological distress and telework jetlag.

		Psychological Distress; *n* (%)		
	Total	NHPD	HPD	*p*-Value
Telework jetlag				<0.001 *
<1 h	1557	1343 (86.3)	214 (13.7)	
≥1 h	232	181 (78.0)	51 (22.0)	

**Abbreviations**: NHPD: non-high psychological distress; HPD: high psychological distress. * Significant difference in χ^2^ test.

**Table 5:** Multivariate regression analysis including variables related to telework and psychological distress.

		Model 1		Model 2		Model 3		Model 4	
		OR	95%CI	OR	95%CI	OR	95%CI	OR	95%CI
Telework jetlag									
<1 h		1.00		1.00		1.00		1.00	
≥1 h		1.82	1.26–2.62	1.68	1.13–2.49	1.73	1.14–2.63	1.80	1.16–2.79

**Abbreviations**: OR: odds ratio; CI: confidence interval. Model 1: adjusted sex, age, living alone, telework frequency, telework location, and telework duration. Model 2: adjusted occupation, employment position, work hours in the past month, job demands, job control, supervisor support, coworker support, and all Model 1 variables. Model 3: adjusted commuting time, means of commuting, exercise habits, alcohol consumption, caffeine consumption, current smoking habits, using electronic terminal outside work, enjoying leisure time, and all Model 2 variables. Model 4: adjusted sleep duration, Athens Insomnia Scale score, and all Model 3 variables.

## Data Availability

The data underlying this article will be shared upon reasonable request from the corresponding authors.

## Supplementary Materials

The following supporting information can be downloaded at https://www.mdpi.com/2624-5175/5/4/40/s1, Table S1: 95%CI and P values of all covariates in the results of multivariate regression analysis.

## References

[1] Wittmann M., Dinich J., Merrow M., Roenneberg T. (2006). Social jetlag: Misalignment of biological and social time. Chronobiol. Int..

[2] Rutters F., Lemmens S.G., Adam T.C., Bremmer M.A., Elders P.J., Nijpels G., Dekker J.M. (2014). Is social jetlag associated with an adverse endocrine, behavioral, and cardiovascular risk profile?. J. Biol. Rhythms.

[3] Roenneberg T., Wirz-Justice A., Merrow M. (2003). Life between clocks: Daily temporal patterns of human chronotypes. J. Biol. Rhythms.

[4] Roenneberg T., Allebrandt K.V., Merrow M., Vetter C. (2012). Social jetlag and obesity. Curr. Biol..

[5] Caliandro R., Streng A.A., van Kerkhof L.W.M., van der Horst G.T.J., Chaves I. (2021). Social jetlag and related risks for human health: A timely review. Nutrients.

[6] Islam Z., Akter S., Kochi T., Hu H., Eguchi M., Yamaguchi M., Kuwahara K., Kabe I., Mizoue T. (2018). Association of social jetlag with metabolic syndrome among Japanese working population: The Furukawa Nutrition and Health Study. Sleep Med..

[7] Hu L., Harper A., Heer E., McNeil J., Cao C., Park Y., Martell K., Gotto G., Shen-Tu G., Peters C. (2020). Social jetlag and prostate cancer incidence in Alberta’s Tomorrow Project: A prospective cohort study. Cancers.

[8] Islam Z., Hu H., Akter S., Kuwahara K., Kochi T., Eguchi M., Kurotani K., Nanri A., Kabe I., Mizoue T. (2020). Social jetlag is associated with an increased likelihood of having depressive symptoms among the Japanese working population: The Furukawa Nutrition and Health Study. Sleep.

[9] Mota M.C., Silva C.M., Balieiro L.C.T., Fahmy W.M., Marqueze E.C., Moreno C.R.C., Crispim C.A. (2021). Social jetlag is associated with impaired metabolic control during a 1-year follow-up. Front. Physiol..

[10] Kim J.H., Lyu Y.S., Kim S.Y. (2020). Impact of social jetlag on weight change in adults: Korean National Health and Nutrition Examination Survey 2016–2017. Int. J. Environ. Res. Public Health.

[11] Kelly R.M., McDermott J.H., Coogan A.N. (2020). Differences in sleep offset timing between weekdays and weekends in 79,161 Adult Participants in the UK Biobank. Clocks Sleep.

[12] Scheer F.A., Hilton M.F., Mantzoros C.S., Shea S.A. (2009). Adverse metabolic and cardiovascular consequences of circadian misalignment. Proc. Natl. Acad. Sci. USA.

[13] Archer S.N., Laing E.E., Möller-Levet C.S., van der Veen D.R., Bucca G., Lazar A.S., Santhi N., Slak A., Kabiljo R., von Schantz M. (2014). Mistimed sleep disrupts circadian regulation of the human transcriptome. Proc. Natl. Acad. Sci. USA.

[14] Wehr T.A., Wirz-Justice A., Goodwin F.K., Duncan W., Gillin J.C. (1979). Phase advance of the circadian sleep-wake cycle as an antidepressant. Science.

[15] Korman M., Tkachev V., Reis C., Komada Y., Kitamura S., Gubin D., Kumar V., Roenneberg T. (2020). COVID-19-mandated social restrictions unveil the impact of social time pressure on sleep and body clock. Sci. Rep..

[16] Leone M.J., Sigman M., Golombek D.A. (2020). Effects of lockdown on human sleep and chronotype during the COVID-19 pandemic. Curr. Biol..

[17] Blume C., Schmidt M.H., Cajochen C. (2020). Effects of the COVID-19 lockdown on human sleep and rest–activity rhythms. Curr. Biol..

[18] Bottary R., Fields E.C., Ugheoke L., Denis D., Mullington J.M., Cunningham T.J. (2022). Changes in sleep regularity and perceived life stress across the COVID-19 Pandemic: A Longitudinal Analysis of a Predominately Female United States Convenience Sample. Clocks Sleep.

[19] Benedict C., Brandão L.E.M., Merikanto I., Partinen M., Bjorvatn B., Cedernaes J. (2021). Meal and sleep timing before and during the COVID-19 pandemic: A cross-sectional anonymous survey study from Sweden. Clocks Sleep.

[20] Knight C., Olaru D., Lee J., Parker S. (2022). The Loneliness of the Hybrid Worker. MIT Sloan Management Review. https://sloanreview.mit.edu/article/the-loneliness-of-the-hybrid-worker/.

[21] Buomprisco G., Ricci S., Perri R., De Sio S. (2021). Health and telework: New challenges after COVID-19 pandemic. Eur. J. Public Health.

[22] Tavares A.I. (2017). Telework and health effects review. Int. J. Health Care.

[23] Vander Elst T., Verhoogen R., Sercu M., Van den Broeck A., Baillien E., Godderis L. (2017). Not extent of telecommuting, but job characteristics as proximal predictors of work-related well-being. J. Occup. Environ. Med..

[24] Evanoff B.A., Strickland J.R., Dale A.M., Hayibor L., Page E., Duncan J.G., Kannampallil T., Gray D.L. (2020). Work-related and personal factors associated with mental well-being during the COVID-19 response: Survey of health care and other workers. J. Med. Internet Res..

[25] Mann S., Holdsworth L. (2003). The psychological impact of teleworking: Stress, emotions and health. New Technol. Work Employ..

[26] Niu Q., Nagata T., Fukutani N., Tezuka M., Shimoura K., Nagai-Tanima M., Aoyama T. (2021). Health effects of immediate telework introduction during the COVID-19 era in Japan: A cross-sectional study. PLoS ONE.

[27] Martin L., Hauret L., Fuhrer C. (2022). Digitally transformed home office impacts on job satisfaction, job stress and job productivity. COVID-19 findings. PLoS ONE.

[28] Carillo K., Cachat-Rosset G., Marsan J., Saba T., Klarsfeld A. (2021). Adjusting to epidemic-induced telework: Empirical insights from teleworkers in France. Eur. J. Inf. Syst..

[29] Anderson A.J., Kaplan S.A., Vega R.P. (2015). The impact of telework on emotional experience: When, and for whom, does telework improve daily affective well-being?. Eur. J. Work Organ. Psychol..

[30] Bailey D.E., Kurland N.B. (2002). A review of telework research: Findings, new directions, and lessons for the study of modern work. J. Organ. Behav..

[31] Shin B., El Sawy O.A., Sheng O.R.L., Higa K. (2000). Telework: Existing research and future directions. J. Organ Comput. Electron. Commer.

[32] Chirico F., Zaffina S., Di Prinzio R.R., Girogi G., Ferrari G., Capitanelli I., Sacco A., Szarpak L., Nucera G., Taino G. (2021). Working from home in the context of COVID-19: A systematic review of physical and mental health effects on teleworkers. J. Health Soc. Behav..

[33] Moglia M., Hopkins J., Bardoel A. (2021). Telework, hybrid work and the United Nation’s Sustainable Development Goals: Towards policy coherence. Sustainability.

[34] Ministry of Health, Labour and Welfare. Comprehensive Survey of Living Conditions in 2019. https://www.mhlw.go.jp/toukei/saikin/hw/k-tyosa/k-tyosa19/index.html.

[35] Breslau N., Peterson E.L., Schultz L.R., Chilcoat H.D., Andreski P. (1998). Major depression and stages of smoking. A longitudinal investigation. Arch Gen Psychiatry.

[36] Churchill S.A., Farrell L. (2017). Alcohol and depression: Evidence from the 2014 health survey for England. Drug Alcohol Depend..

[37] Gordon B.R., McDowell C.P., Lyons M., Herring M.P. (2017). The effects of resistance exercise training on anxiety: A meta-analysis and meta-regression analysis of randomized controlled trials. Sports Med..

[38] Roenneberg T., Kuehnle T., Juda M., Kantermann T., Allebrandt K., Gordijn M., Merrow M. (2007). Epidemiology of the human circadian clock. Sleep Med. Rev..

[39] Roenneberg T., Pilz L.K., Zerbini G., Winnebeck E.C. (2019). Chronotype and social jetlag: A (self-) critical review. Biology.

[40] Jankowski K.S. (2017). Social jet lag: Sleep-corrected formula. Chronobiol. Int..

[41] Okajima I., Komada Y., Ito W., Inoue Y. (2021). Sleep debt and social jetlag associated with sleepiness, mood, and work performance among workers in Japan. Int. J. Environ. Res. Public. Health.

[42] Taylor A., Wright H.R., Lack L.C. (2008). Sleeping-in on the weekend delays circadian phase and increases sleepiness the following week. Sleep Biol. Rhythm..

[43] Lee A., Myung S.K., Cho J.J., Jung Y.J., Yoon J.L., Kim M.Y. (2017). Night shift work and risk of depression: Meta-analysis of observational studies. J. Korean Med. Sci..

[44] Roenneberg T., Kuehnle T., Pramstaller P.P., Ricken J., Havel M., Guth A., Merrow M. (2004). A marker for the end of adolescence. Curr. Biol..

[45] Germain A., Kupfer D.J. (2008). Circadian rhythm disturbances in depression. Hum. Psychopharmacol..

[46] Salgado-Delgado R., Tapia Osorio A., Saderi N., Escobar C. (2011). Disruption of circadian rhythms: A crucial factor in the etiology of depression. Depress. Res. Treat..

[47] Morris C.J., Purvis T.E., Hu K., Scheer F.A. (2016). Circadian misalignment increases cardiovascular disease risk factors in humans. Proc. Natl. Acad. Sci. USA.

[48] Moret C., Briley M. (2011). The importance of norepinephrine in depression. Neuropsychiatr. Dis. Treat..

[49] Herbert J. (2013). Cortisol and depression: Three questions for psychiatry. Psychol. Med..

[50] Roenneberg T., Merrow M. (2016). The circadian clock and human health. Curr. Biol..

[51] Kronfeld-Schor N., Visser M.E., Salis L., van Gils J.A. (2017). Chronobiology of interspecific interactions in a changing world. Philos. Trans. R. Soc. Lond. B Biol. Sci..

[52] Souetre E., Salvati E., Wehr T.A., Sack D.A., Krebs B., Darcourt G. (1988). Twenty-four-hour profiles of body temperature and plasma TSH in bipolar patients during depression and during remission and in normal control subjects. Am. J. Psychiatry.

[53] Steiner M., Brown G.M., Goldman S. (1990). Nocturnal melatonin and cortisol secretion in newly admitted psychiatric inpatients. Eur. Arch. Psychiatry Clin. Neurosci..

[54] Peeters F., Berkhof J., Delespaul P., Rottenberg J., Nicolson N.A. (2006). Diurnal mood variation in major depressive disorder. Emotion.

[55] Buhr E.D., Yoo S.H., Takahashi J.S. (2010). Temperature as a universal resetting cue for mammalian circadian oscillators. Science.

[56] McClung C.A. (2007). Circadian genes, rhythms and the biology of mood disorders. Pharmacol. Ther..

[57] Tsang A.H., Barclay J.L., Oster H. (2014). Interactions between endocrine and circadian systems. J. Mol. Endocrinol..

[58] Kessler R.C., Andrews G., Colpe L., Hiripi È., Mroczek D.K., Normand S.-L.T., Walters E.E., Zaslavsky A.M. (2002). Short screening scales to monitor population prevalences and trends in non-specific psychological distress. Psychol. Med..

[59] Furukawa T.A., Kawakami N., Saitoh M., Ono Y., Nakane Y., Nakamura Y., Tachimori H., Iwata N., Uda H., Nakane H. (2008). The performance of the Japanese version of the K6 and K10 in the World Mental Health Survey Japan. Int. J. Methods Psychiatr. Res..

[60] Sakurai K., Nishi A., Kondo K., Yanagida K., Kawakami N. (2011). Screening performance of K6/K10 and other screening instruments for mood and anxiety disorders in Japan. Psychiatry Clin. Neurosci..

[61] Shimomitsu T. (2000). The Final Development of the Brief Job Stress Questionnaire Mainly Used for Assessment of the Individuals. Ministry of Labour Sponsored Grant for the Prevention of Work-Related Illness: The 1999 Report.

[62] Soldatos C.R., Dikeos D.G., Paparrigopoulos T.J. (2000). Athens Insomnia Scale: Validation of an instrument based on ICD-10 criteria. J. Psychosom. Res..

[63] Okajima I., Nakajima S., Kobayashi M., Inoue Y. (2013). Development and validation of the Japanese version of the Athens Insomnia Scale. Psychiatry Clin. Neurosci..

