# Supplementary material for: Relationship between Telework Jetlag and Perceived Psychological Distress among Japanese Hybrid Workers
Source: Clocks Sleep. 2023 Oct 16;5(4):604–14. doi: 10.3390/clockssleep5040040 (PMC10667991; doi:10.3390/clockssleep5040040)
Supplement: Supplementary file 1 [file clockssleep-05-00040-s001.zip › clockssleep-2583616-supplementary.pdf]

Table S1. 95%CI and P values of all covariates in the results of multivariate regression analysis. .

		Model 1		Model 2		Model 3		Model 4	
		95%CI	p-value	95%CI	p-value	95%CI	p-value	95%CI	p-value
Sex			0.680		0.682		0.373		0.19
	Men	Ref		Ref		Ref		Ref	
	Women	0.79-1.43		0.76-1.54		0.81-1.75		0.87-1.98	
Age			0.001*		0.004*		< 0.001*		< 0.001*
	20-29	Ref		Ref		Ref		Ref	
	30-39	0.57-1.29		0.47-1.16		0.42-1.10		0.43-1.17	
	40-49	0.49-1.12		0.46-1.19		0.34-0.97		0.34-1.03	
	50-59	0.33-0.77		0.30-0.82		0.18-0.57		0.18-0.59	
	≧ 60	0.09-0.61		0.07-0.52		0.03-0.29		0.03-0.26	
living alone			0.004*		0.008*		0.019*		0.250
	No	Ref		Ref		Ref		Ref	
	Yes	1.15-2.09		1.12-2.14		1.07-2.17		0.86-1.80	
Telework frequency			0.588		0.901		0.931		0.879
	1-3 d/m	Ref		Ref		Ref		Ref	
	1-2 d/wk	0.58-1.28		0.58-1.36		0.57-1.39		0.65-1.65	
	3-4 d/wk	0.52-1.13		0.56-1.31		0.57-1.36		0.58-1.46	
	≧ 5 d/wk	0.51-1.21		0.52-1.40		0.51-1.43		0.49-1.46	
Teleworking place			0.344		0.634		0.444		0.313
	Living room	Ref		Ref		Ref		Ref	
	Private room	0.60-1.25		0.62-1.36		0.61-1.37		0.53-1.25	
	Bedroom	0.65-1.32		0.65-1.38		0.57-1.25		0.48-1.09	
	Others	0.86-2.31		0.79-2.31		0.80-2.44		0.67-2.19	
Teleworking duration			0.786		0.659		0.730		0.840
	< 1 y	Ref		Ref		Ref		Ref	
	≧ 1 y and < 1.5 y	0.74-1.73		0.78-1.93		0.76-1.93		0.71-1.89	
	≧ 1.5 y	0.69-1.55		0.72-1.71		0.73-1.80		0.69-1.76	
Occupation					0.210		0.187		0.167
	Clerks			Ref		Ref		Ref	
	Engineers			0.77-2.12		0.82-2.34		0.80-2.42	
	Field workers			0.65-3.31		0.62-3.40		0.57-3.35	
	Creators			0.43-1.66		0.38-1.58		0.40-1.77	
	Customer service			0.55-2.75		0.55-2.87		0.48-2.74	
	Sales			0.97-2.48		0.93-2.53		0.97-2.73	
	Others			0.28-1.46		0.27-1.46		0.25-1.41	
Employment position					0.006*		0.015*		0.028*
	Manager or Director			Ref		Ref		Ref	

Chief	0.40-0.98		0.39-0.99		0.42-1.11	
Supervisor	0.31-0.83		0.31-0.89		0.31-0.94	
Regular Employee	0.11-0.76		0.11-0.82		0.09-0.78	
Work hours past 1 month		0.144		0.149		0.147
< 160 h/m	Ref		Ref		Ref	
≧ 160 and < 200 h/m	0.52-1.02		0.51-1.02		0.49-1.03	
≧ 200 h/m	0.60-1.44		0.58-1.44		0.58-1.49	
Job stressors and social supports						
Job demands	1.08-1.29	< 0.001*	1.07-1.27	0.001*	1.03-1.24	0.011*
Job control	0.67-0.81	< 0.001*	0.69-0.83	< 0.001*	0.7-0.85	< 0.001*
Supervisor support	0.85-1.01	0.089	0.84-1.01	0.072	0.86-1.03	0.196
Coworker support	0.80-0.95	0.002*	0.82-0.99	0.023*	0.84-1.02	0.112
Commuting time				0.163		0.115
< 60 min			Ref		Ref	
≧ 60min			0.90-1.85		0.93-1.98	
Means of commuting				0.040*		0.208
Crawded train			Ref		Ref	
Uncrawded train			0.50-1.51		0.56-1.81	
On foot			0.51-1.40		0.57-1.65	
Others			0.97-3.32		0.90-3.30	
Exercise habits				0.851		0..838
Not physically active and no intention to change			Ref		Ref	
Not active but intending to change			0.72-2.10		0.70-2.15	
Doing some activity but not enogh			0.68-1.94		0.60-1.79	
Regularly active but not the habit			0.72-2.52		0.68-2.52	
Regularly active in the habit			0.56-1.95		0.57-2.12	
Drinking alcohol				0.090		0.026*
Never or rarely			Ref		Ref	
less than 2 d/wk			0.63-1.32		0.60-1.30	
3-6 d/wk			0.35-0.88		0.30-0.78	
everyday			0.51-1.29		0.44-1.16	
Taking caffeine				0.542		0.57
Never or rarely			Ref		Ref	
2-6 d/wk			0.52-1.70		0.51-1.77	

Everyday but less than twice a day	0.51-1.52		0.54-1.69	
Everyday and more than three times a day	0.65-2.05		0.67-2.26	
Current smoking		0.044*		0.031*
No	Ref		Ref	
Yes	1.01-2.04		1.04-2.19	
Using electronic terminal outside work		0.598		0.087
< 2 h/d	Ref		Ref	
≧ 2 h/d	0.67-1.26		0.54-1.04	
Enjoying leisure time		< 0.001*		< 0.001*
Yes	Ref		Ref	
No	2.17-4.09		1.80-3.51	
Sleep duration				0.002*
≧ 7 h			Ref	
< 7 h			0.41-0.82	
AIS score				< 0.001*
< 6			Ref	
≧ 6			3.70-7.31	

Abbreviations: CI: confidence interval, Ref: reference, AIS: Athens Insomnia Scale.

* Significant difference by multiple logistic regression.
